# Recurrent Immature Teratoma Mimicking Growing Teratoma Syndrome Following Initial Resection: A Diagnostic Pitfall

**DOI:** 10.1155/crog/4866935

**Published:** 2025-10-28

**Authors:** Shogo Nishino, Hidetaka Nomura, Takato Goto, Ryo Nimura, Yoichi Aoki, Sanshiro Okamoto, Makiko Omi, Yui Kojima, Akiko Tonooka, Hiroyuki Kanao

**Affiliations:** ^1^Department of Gynecologic Oncology, The Cancer Institute Hospital of Japanese Foundation for Cancer Research, Tokyo, Japan; ^2^Division of Pathology, The Cancer Institute Hospital of Japanese Foundation for Cancer Research, Tokyo, Japan; ^3^Department of Pathology, Clinicopathology Center, The Cancer Institute Hospital of Japanese Foundation for Cancer Research, Tokyo, Japan

**Keywords:** germ cell tumors, growing teratoma syndrome, histopathology, immature teratoma, ovarian cancer

## Abstract

**Background:**

Growing teratoma syndrome (GTS) is a rare condition characterized by the enlargement of metastatic masses during or after chemotherapy for malignant germ cell tumors (GCTs), despite normalized tumor marker levels. It is defined by three criteria: (1) enlarging or new masses during or after chemotherapy, (2) normal tumor marker levels, and (3) histological presence of only mature teratoma elements. Differentiating GTS from recurrent immature teratoma is challenging, as both conditions may present similarly in imaging and tumor marker profiles.

**Case Presentation:**

We report the case of a 20-year-old woman diagnosed with a mixed ovarian GCT, consisting of grade 2 immature teratoma and yolk sac tumor. After undergoing right salpingo-oophorectomy and chemotherapy, she remained in remission for 4 years. Follow-up imaging revealed enlarged para-aortic lymph nodes. Due to normal tumor markers and a lack of response to chemotherapy, the condition was initially diagnosed as GTS, prompting surgical resection of the lymph nodes. Histopathological analysis, however, revealed immature neuroepithelial elements consistent with grade 3 immature teratoma, contradicting the GTS diagnosis. The final diagnosis was revised to recurrent immature teratoma.

**Conclusion:**

This case highlights the diagnostic challenges in distinguishing between GTS and recurrent immature teratoma. While normal tumor markers and tumor growth following chemotherapy may suggest GTS, histopathological confirmation is essential. Clinicians should maintain a high index of suspicion for recurrent immature teratoma in cases mimicking GTS and consider surgical resection for definitive diagnosis. Multidisciplinary evaluation remains crucial in the management of ovarian GCTs.

## 1. Introduction

Growing teratoma syndrome (GTS) is a rare condition characterized by the enlargement of metastatic masses during or after chemotherapy for malignant germ cell tumors (GCTs), despite normalization of tumor markers [1]. It is defined by three diagnostic criteria: (1) the appearance of enlarging or new masses during or after chemotherapy, (2) normal tumor marker levels, and (3) the presence of only mature teratoma components on histological examination [2–4]. Although GTS has been described in the literature, its rarity and atypical presentation can make diagnosis challenging, especially when it mimics tumor recurrence or progression.

Here, we report a case initially diagnosed as GTS but later confirmed to be a recurrent immature teratoma. This case underscores the diagnostic challenges in differentiating between GTS and recurrent immature teratoma and highlights the importance of histopathological confirmation and vigilant clinical follow-up in patients with GCTs.

## 2. Case Presentation

**Figure 1:**
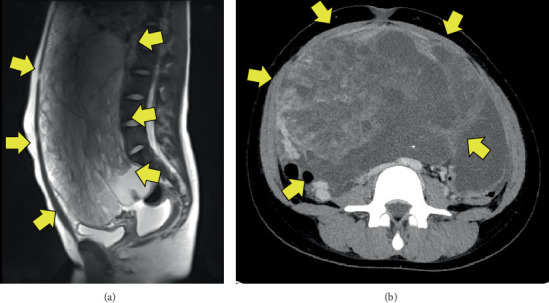
(a) Sagittal section of magnetic resonance imaging showing a giant abdominopelvic mass (primary ovarian tumor). (b) Axial computed tomography image demonstrating the same mass.

A 20-year-old woman presented with a history of a mixed germ cell tumor (GCT) of the right ovary diagnosed 4 years earlier. She underwent initial surgery and adjuvant chemotherapy at the referring institution. At the time of diagnosis, her serum alpha-fetoprotein (AFP) was 71,171.0 ng/mL (normal: < 10 ng/mL) and cancer antigen 125 (CA125) was 508.1 U/mL (normal: < 35 U/mL). She underwent right salpingo-oophorectomy and omentectomy for a large right ovarian tumor. Histopathological examination confirmed a mixed GCT consisting of 70% grade 2 immature teratoma and 30% yolk sac tumor, graded according to the criteria proposed by O'Connor and Norris et al. [5] (Figure 1). The tumor was staged as FIGO stage IC2 [6]. Postoperatively, she received three cycles of adjuvant chemotherapy with bleomycin, etoposide, and cisplatin (BEP). Her tumor markers normalized with treatment, and she was regularly followed on an outpatient basis.

**Figure 2:**
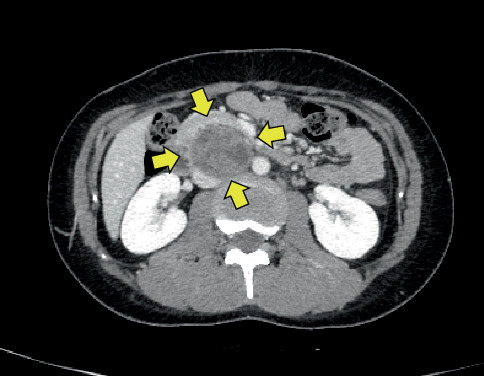
Computed tomography image showing enlarged para-aortic lymph nodes, the largest measuring 5.5 cm in diameter.

Four years later, abdominal and pelvic computed tomography (CT) revealed enlarged para-aortic lymph nodes. Fluorodeoxyglucose positron emission tomography/CT (FDG-PET/CT) showed increased uptake in the same region, with a maximum standardized uptake value (SUVmax) of 12.54, suggesting recurrence of malignant GCT. She received one cycle of BEP without tumor shrinkage, followed by two cycles of paclitaxel, ifosfamide, and cisplatin, which also failed to reduce the tumor size. Due to the lack of response to chemotherapy and normal tumor marker levels, the diagnosis was revised to growing teratoma syndrome (GTS). At that time, her AFP was 3.1 ng/mL and CA125 was 5.4 U/mL, both within normal ranges. hCG and LDH were not evaluated initially because the diagnosis of immature teratoma was not suspected at the referring institution. After completing these treatments at the referring institution, she was referred to our hospital for further evaluation and surgical management. CT imaging revealed two enlarged para-aortic lymph nodes, the largest measuring 5.5 cm in diameter (Figure 2). The tumor was deemed chemotherapy-resistant but surgically resectable, and a laparotomy was performed for complete resection.

**Figure 3:**
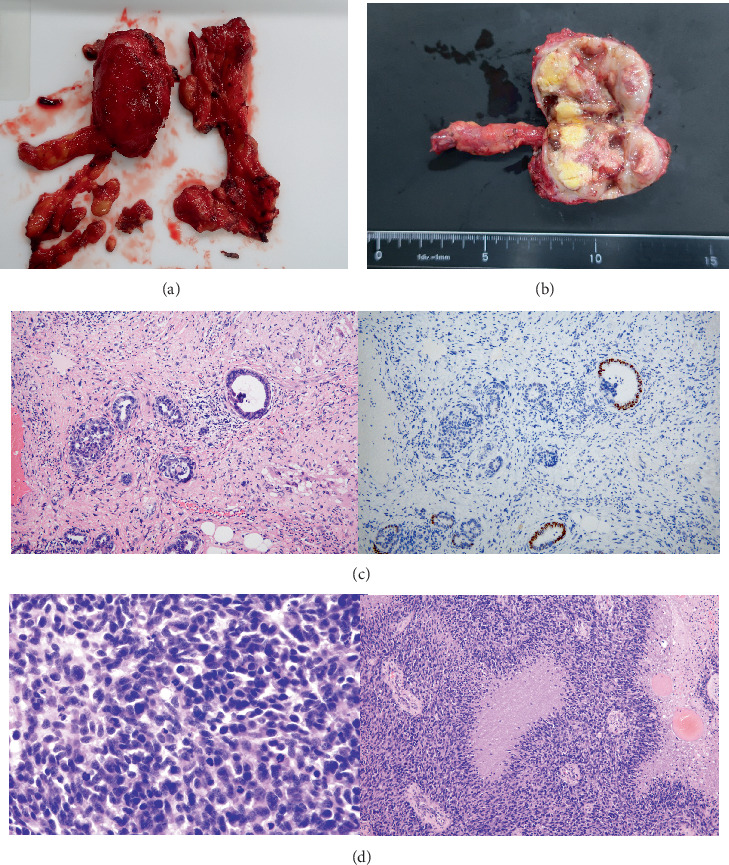
(a) Gross photograph of the resected para-aortic lymph node. (b) Cut surface of the para-aortic lymph node. A tubular structure identified intraoperatively corresponds to the right ovarian vein. Although yellowish areas were observed macroscopically, no yolk sac tumor components were identified histologically. (c) Hematoxylin and eosin (H&E) staining (left, × 200) shows glandular structures composed of columnar epithelium with a high nuclear-to-cytoplasmic ratio. Immunohistochemistry for SALL4 (right, × 200) demonstrates focal nuclear positivity. (d) H&E staining (left, × 200) reveals densely proliferating immature cells with a high nuclear-to-cytoplasmic ratio and oval to spindle-shaped nuclei. The tumor shows pseudopalisading patterns and geographic coagulative necrosis (right, × 100).

Intraoperatively, masses were found in the para-aortic lymph nodes, and 25 lymph nodes were removed with complete resection achieved. Histopathological analysis revealed immature neuroepithelial elements consistent with grade 3 immature teratoma in 3 of the 25 lymph nodes, without any yolk sac tumor components [5] (Figure 3). These findings were incompatible with the diagnosis of GTS, which is defined by the presence of only mature teratoma elements. Therefore, the final diagnosis was revised from clinically suspected GTS to recurrent immature teratoma, based on histopathological confirmation. Postoperatively, the patient was followed at the referring institution. There were no postoperative complications. However, 4 months after surgery, multiple lymph node metastases were identified, and she subsequently underwent palliative radiotherapy.

## 3. Discussion

This case report highlights two key findings: first, the diagnostic challenge in differentiating GTS from recurrent immature teratoma (IT), and second, that recurrence of IT may present with normal tumor markers, mimicking GTS.

Both GTS and recurrent immature teratoma can present as enlarging masses with normal tumor marker levels. GCTs are a heterogeneous group of neoplasms that arise in both gonadal and extragonadal locations. Immature teratomas are a significant subset, particularly in young women. Chemotherapy has greatly improved outcomes in these patients. GTS, although rare, occurs in 1.9%–7.6% of non-seminomatous GCTs [7] and is characterized by tumor enlargement during or after chemotherapy in the presence of normalized tumor markers [1]. First described by Logothetis et al. [4], GTS remains a diagnostic challenge. While recurrent immature teratomas typically present with elevated tumor markers, there are documented cases of recurrence without marker elevation, as in our case [8].

Thus, two of the criteria for GTS—tumor enlargement during or after chemotherapy and normal tumor markers—can also be observed in recurrent immature teratomas.

Previous imaging studies of GTS have reported features such as increasing lesion size, a growing cystic component, well-circumscribed margins without invasion of surrounding structures, internal septations, calcifications, and increased fat content [3, 9]. However, these features are not exclusive to GTS and can overlap with findings in recurrent immature teratomas.

Therefore, clinical progression and imaging findings alone are insufficient for differentiating GTS from recurrent immature teratoma. Even with close surveillance, the two conditions can appear similar—especially since malignant recurrence may occur without elevated tumor markers, and GTS can show FDG uptake on PET/CT [10]. In our case, the SUVmax was 12.54, which is consistent with values seen in malignant germ cell tumors; however, similar levels of uptake have also been reported in GTS, highlighting the limited specificity of FDG-PET/CT in this context [10].

In the present case, all criteria for GTS were seemingly met: The patient had enlarging para-aortic lymph nodes during follow-up after chemotherapy and normal tumor marker levels. However, histopathological analysis revealed immature neuroepithelial elements, which were inconsistent with the initial clinical impression of GTS and led to a revised diagnosis of recurrent immature teratoma following pathological confirmation. Immunohistochemical analysis provided critical diagnostic clarity. In particular, nuclear positivity for SALL4 supported the presence of immature germ cell components, helping to distinguish recurrent IT from GTS, which lacks such expression. This case underscores the crucial role of surgical resection and comprehensive pathological evaluation in establishing an accurate diagnosis. Differentiating recurrent IT from GTS is not only diagnostically important but also carries therapeutic implications. While GTS is managed primarily through surgical excision with excellent prognosis, recurrent IT may require additional systemic chemotherapy depending on histological grade and disease extent. Misdiagnosis may result in overtreatment or undertreatment, underscoring the need for accurate pathological confirmation.

A multidisciplinary approach and high clinical suspicion are essential in managing patients with a history of GCTs. Serial imaging during and after chemotherapy is vital to detect changes in tumor size and characteristics early. When GTS is suspected, surgical resection remains the gold standard for both diagnosis and treatment.

This study has several limitations. First, it reports a single case, limiting the generalizability of the findings. Second, long-term follow-up data are not yet available, which would offer valuable insights into prognosis and potential recurrence. These limitations highlight the need for larger-scale studies to improve understanding of the diagnostic challenges and optimal management of recurrent immature teratoma and GTS.

Ultimately, while GTS is a recognized entity, diagnosis should not be made without histopathological confirmation. Clinicians should remain vigilant for the possibility of recurrent immature teratoma in cases mimicking GTS. Long-term follow-up studies are needed to better define the clinical course and guide treatment strategies for both conditions. This report contributes to the evolving understanding of GCT recurrence and the complexities of diagnosis and management in these cases.

## Data Availability

The data supporting the findings of this study are available from the corresponding author upon reasonable request.
